# Probing Coherent Vibrations of Organic Phosphonate Radical Cations with Femtosecond Time-Resolved Mass Spectrometry

**DOI:** 10.3390/molecules24030509

**Published:** 2019-01-31

**Authors:** Derrick Ampadu Boateng, Mi’Kayla D. Word, Katharine Moore Tibbetts

**Affiliations:** Department of Chemistry, Virginia Commonwealth University, Richmond, VA 23284, USA; ampaduboatend@vcu.edu (D.A.B.); wordmd@vcu.edu (M.D.W.)

**Keywords:** femtosecond spectroscopy, pump–probe, mass spectrometry, wave packet dynamics, organic phosphonates

## Abstract

Organic phosphates and phosphonates are present in a number of cellular components that can be damaged by exposure to ionizing radiation. This work reports femtosecond time-resolved mass spectrometry (FTRMS) studies of three organic phosphonate radical cations that model the DNA sugar-phosphate backbone: dimethyl methylphosphonate (DMMP), diethyl methylphosphonate (DEMP), and diisopropyl methylphosphonate (DIMP). Upon ionization, each molecular radical cation exhibits unique oscillatory dynamics in its ion yields resulting from coherent vibrational excitation. DMMP has particularly well-resolved 45 fs (732±28 cm${}^{-1}$) oscillations with a weak feature at 610–650 cm${}^{-1}$, while DIMP exhibits bimodal oscillations with a period of ∼55 fs and two frequency features at 554±28 and 670–720 cm${}^{-1}$. In contrast, the oscillations in DEMP decay too rapidly for effective resolution. The low- and high-frequency oscillations in DMMP and DIMP are assigned to coherent excitation of the symmetric O–P–O bend and P–C stretch, respectively. The observation of the same ionization-induced coherently excited vibrations in related molecules suggests a possible common excitation pathway in ionized organophosphorus compounds of biological relevance, while the distinct oscillatory dynamics in each molecule points to the potential use of FTRMS to distinguish among fragment ions produced by related molecules.

## 1. Introduction

Organic phosphates and phosphonates comprise important cellular components including the DNA backbone, lipid membranes, and post-translationally modified proteins. The phosphate group in DNA is a primary target of radiation-induced damage, where one-electron oxidation of the phosphate results in formation of sugar radicals that induce lesions such as single- and double-strand breaks [1]. Due to the importance of understanding the dynamics and chemical mechanisms leading to DNA damage upon one-electron oxidation of the sugar-phosphate backbone, many experimental techniques have been applied for this purpose. For instance, experiments conducted at cryogenic temperatures have identified the structures of sugar radicals formed by one-electron phosphate oxidation in γ-irradiated DNA [2]. Picosecond time-resolved electronic spectroscopy of nucleotides irradiated with 5 ps electron pulses has determined lifetimes of phosphate radicals and timescales of backbone-to-base hole transfer [3,4]. Mass spectrometry is also widely used to characterize the products of radiation-induced DNA damage including modified sugars and bases [5]. While these studies provide significant insight into how one-electron oxidation of the sugar-phosphate backbone leads to DNA damage, little is known about how vibrational excitations in oxidized phosphates may influence reaction mechanisms leading to DNA damage.

Femtosecond time-resolved mass spectrometry (FTRMS) is uniquely suited to resolve vibrational excitations in molecular radical cations such as one-electron oxidized organic phosphates. FTRMS of radical cations is a pump–probe technique that involves: (1) ionization of the target molecule with an intense fs laser “pump” pulse to produce the molecular cation, which is followed by (2) excitation of the cation with a weaker “probe” pulse that induces dissociation. The resulting fragment ions are then detected in a time-of-flight mass spectrometer. The exceptionally short duration and broad bandwidth of the ionizing pump pulse results in the simultaneous population of multiple excited vibrational states with accessible Franck–Condon factors in the cation, which creates a coherent superposition, or vibrational “wave packet” [6]. Tracking the subsequent ultrafast dynamics of the wave packet with probe excitation has revealed how coherent vibrational excitation leads to bond dissociation in a number of polyatomic radical cations [7,8,9,10,11,12,13,14,15,16,17,18,19]. While FTRMS measurements are inherently in the gas phase, the fundamental insights into molecular dissociation dynamics of molecules independent of solvent effects provide a baseline for comparison to solution-phase studies, as noted in recent reviews of DNA electronic excited-state dynamics measurements with FTRMS [20,21].

**Scheme 1:**
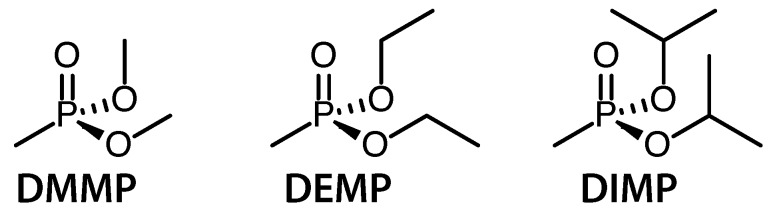
Structures of molecules investigated in this work.

Recently, we reported on coherent vibrational dynamics in the radical cation of dimethyl methylphosphonate (DMMP), a model of the DNA sugar-phosphate backbone [18]. In this work, we compare the vibrational dynamics in DMMP to the related molecules diethyl methylphosphonate (DEMP) and diisopropyl methylphosphonate (DIMP) (Scheme 1). The methylphosphonates under investigation model not only the DNA sugar-phosphate backbone, but also an important class of phosphonate natural products that have antibacterial and other interesting properties [22]. Although the present study considered neutral molecules instead of the anions present under biological conditions, formation of radical cations in our experiments can be considered in analogy to the formation of neutral radicals via one-electron oxidation in biological systems. The similarities and differences in the coherent vibrational dynamics across this molecular series reveal important information about how small changes in molecular structure can influence the ionization-induced vibrational dynamics and subsequent bond-dissociation reactions. These results not only provide insight into the potential contributions of vibrational excitation to ionization-induced reactions of biologically relevant organophosphorus compounds, but also raise the possibility of distinguishing between similar molecules with mass spectrometry through the unique vibrational signatures reflected in their fragment ions.

## 2. Results

This section presents FTRMS results on DMMP, DEMP, and DIMP (Section 2.1) indicating that coherent vibrational motion is excited upon electron removal to form the respective radical cations. All measurements were taken with 1500 nm, 18 fs, $1.5\times 10^{14}$ W cm${}^{-2}$ ionizing pump pulses and 800 nm, 25 fs, $1.5\times 10^{13}$ W cm${}^{-2}$ probe pulses (see Section 4.2 for details). The coherently excited vibrational mode(s) in each molecule were assigned using DFT calculations of each radical cation (Section 2.2).

### 2.1. Femtosecond Time-Resolved Mass Spectrometry (FTRMS)

**Figure 1:**
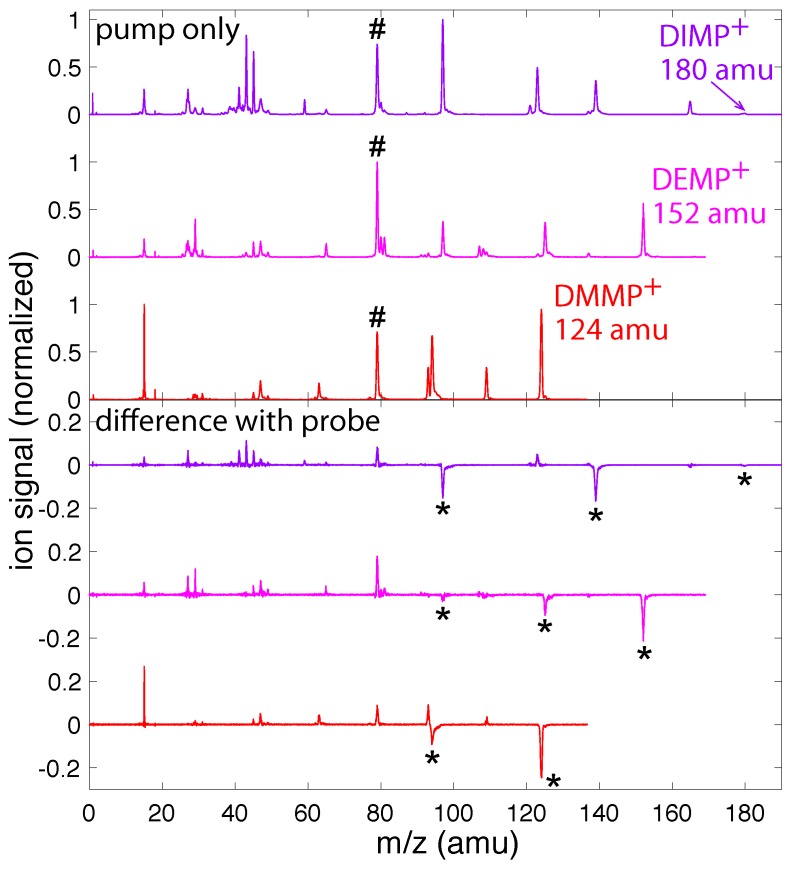
(**Top**) Mass spectra of DMMP (red), DEMP (magenta), and DIMP (violet) taken with only the pump pulse; and (**Bottom**) difference between pump-only mass spectra and mass spectra taken with the probe pulse at a delay of τ=100 fs.

Figure 1 (top) shows the mass spectra of DMMP (red), DEMP (magenta), and DIMP (violet) taken with only the 1500 nm pump pulse. DMMP and DEMP show a prominent parent molecular ion signal, while only a small amount of parent ion is present in DIMP. Each molecule produced a series of fragment ions, some of which (e.g., *m*/*z* = 79, marked with a #) were observed in all three molecules. Significant changes in the fragmentation pattern of each molecule were observed when the 800 nm probe pulse arrived at a delay of τ=100 fs after the ionizing pump pulse. In particular, the yield of each parent ion and large fragment ions marked with a ⋆ decreased, while the yield of smaller fragment ions increased. By measuring mass spectra of each molecule while scanning the pump–probe delay, the transient yields of the parent and dominant fragment ions as a function of delay τ could be extracted.

**Figure 2:**
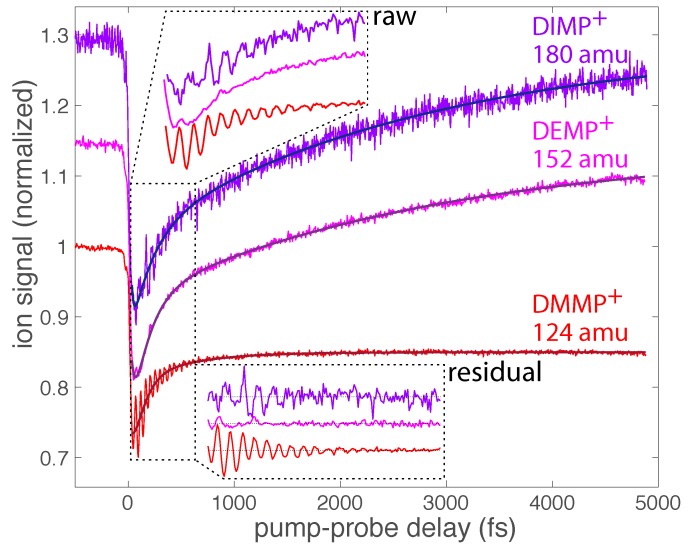
Transient ion signals of the parent molecular ions of DMMP (red), DEMP (magenta), and DIMP (violet) as a function of pump–probe delay. The region between 30 and 700 fs is magnified in the insets to show the oscillations. The dark solid lines denote fits to Equation (1).

Figure 2 shows the transient yields of the DMMP, DEMP, and DIMP parent molecular ions as a function of pump–probe delay. The yields were obtained from the integrated intensity over the corresponding mass spectral peaks (*m*/*z* = 124 for DMMP, *m*/*z* = 152 for DEMP, and *m*/*z* = 180 for DIMP). All signals were normalized to unity at negative delays, and the signals of DEMP and DIMP were shifted on the ordinate axis for clarity. While the most salient feature of each transient ion signal is its slow decay dynamics over the 5000 fs measurement window, embedded in these signals are ultrafast oscillations in the yield signals. These oscillations in the raw ion signals are present over the first ∼700 fs after ionization, as seen in the magnified inset labeled “raw”. Oscillating ion yields with pump–probe delay signify vibrational wave packet dynamics in radical cations, and the frequency of these oscillations specifies the vibrational mode that is coherently excited during ionization [7,8,9,10,11,12,13,14,15,16,17,18,19].

To isolate the oscillations in the transient ion signals for further analysis, the slow decay dynamics may be fit to a multiexponential decay function,

(1) $$S\left(\tau \right)=a\exp\left(-\tau /T_{1}\right)+b\exp\left(-\tau /T_{2}\right)+c\exp\left(-\tau /T_{3}\right)+d$$

where $T_{1}$, $T_{2}$, and $T_{3}$ denote dynamical timescales, and the constants *a*–*c* the corresponding relative amplitudes. The constant *d* corresponds to the asymptotic ion signal as the delay τ→∞. The dynamical timescales may correspond to processes including geometric relaxation, excited state lifetimes, and intramolecular rearrangement reactions. Assignment of these timescales was not the goal of the present work; instead we focused on the residual oscillatory signals remaining after subtraction of the dynamics described by Equation (1). The nonlinear least squares fits of each transient signal in Figure 2 to Equation (1) are shown as the dark solid lines. The residual oscillatory ion signals that remain after subtraction of Equation (1) make the oscillations clearer, as shown in the inset labeled “residual”. In the following sections on DMMP, DEMP, and DIMP, the oscillatory parent transient signals are compared with those of fragment ions in each molecule that exhibit oscillations.

#### 2.1.1. DMMP

**Figure 3:**
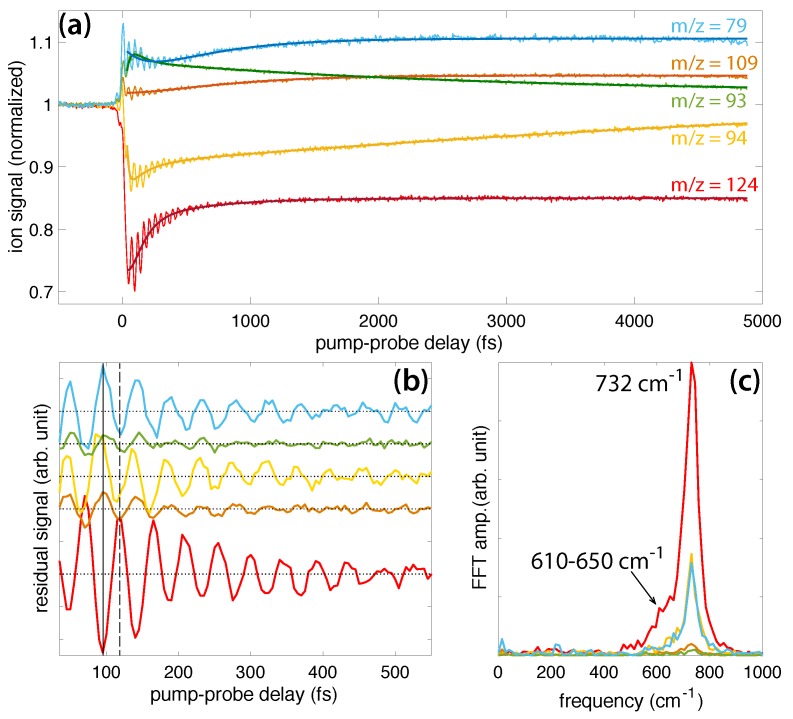
(**a**) Transient signals of DMMP${}^{\cdot +}$ (*m*/*z* = 124, red), PO_3_(CH_3_)_3_^+^ (*m*/*z* = 109, orange), PO_2_C_2_H_7_^+^ (*m*/*z* = 94, gold), PO_2_(CH_3_)_2_^+^ (*m*/*z* = 93, green), and PO_2_CH_4_^+^ (*m*/*z* = 79, blue). The dark solid lines denote fits to Equation (1). (**b**) Residual ion signals after exponential dynamics are subtracted. Dotted horizontal lines denote zero residual for each signal. Solid and dashed vertical lines denote the first minimum and maximum of the parent ion yield, respectively. (**c**) FFT of residual ion signals in (**b**).

Figure 3a shows the transient ion signals of DMMP that exhibit oscillations: parent molecular ion DMMP${}^{\cdot +}$ (*m*/*z* = 124, red), PO_3_(CH_3_)_3_^+^ (*m*/*z* = 109, orange), PO_2_C_2_H_7_^+^ (*m*/*z* = 94, gold), PO_2_(CH_3_)_2_^+^ (*m*/*z* = 93, green), and PO_2_CH_4_^+^ (*m*/*z* = 79, blue). All ion signals were normalized to the parent ion yield at negative delay and shifted on the ordinate axis to unity at negative delay to illustrate the relative change in each signal upon interaction with the probe pulse. At positive delays, the depletion of the DMMP${}^{\cdot +}$ and PO_2_C_2_H_7_^+^ signals and enhancement of the PO_3_(CH_3_)_3_^+^, PO_3_(CH_3_)_2_^+^, and PO_2_CH_4_^+^ signals indicate that the probe pulse induced formation of the latter enhanced species via excitation of the depleted DMMP${}^{\cdot +}$ [7,8,9,10,11,12,13,14,15,16,17,18,19]. The dark solid lines denote fits to Equation (1), and fitting coefficients for each signal are provided in the Supplementary Material (Table S1). The residual ion signals upon subtraction of Equation (1) are shown in Figure 3b. The first maximum and minimum of the parent signal are indicated by the dashed and solid lines, respectively. All fragment ion signals oscillate approximately antiphase with the parent signal; they exhibit a maximum at the solid line and a minimum at the dashed line. The antiphase oscillations indicate that these dissociation products are formed upon probe pulse excitation when the vibrational wave packet passes through a geometry with strong dipole coupling to one or more excited states that are accessible by the probe photon energy [7,8,9,10,11,12,13,14,15,16,17,18,19]. The oscillation period therefore determines the vibrational frequency of the coherently excited normal mode, which can be obtained by Fast Fourier Transform (FFT) of the time-dependent residual ion signals. FFT was performed over the sampling window of 35–2500 fs with 5 fs resolution, yielding a frequency resolution of 14 cm${}^{-1}$. The FFT frequency spectra (Figure 3c) exhibit a strong peak at 732±28 cm${}^{-1}$ (error estimated by the peak full width at half maximum). This peak is slightly down-shifted from the 750 cm${}^{-1}$ frequency reported in Ref. [18], although the observed frequencies agree to within the estimated error. The DMMP${}^{\cdot +}$ spectrum also contains a distinct shoulder in the range of 610–650 cm${}^{-1}$ that was not visible in the previous investigation, likely because the slow exponential dynamics were not subtracted off prior to implementing the FFT or because the longer sampling window in the present work results in better frequency resolution. This shoulder peak may be attributed to an additional vibrational excitation not previously reported, as discussed below in Section 2.2. The lower intensity of the shoulder peak suggests that its associated mode has a lower probability of being excited than the mode associated with the main peak.

**Scheme 2:**
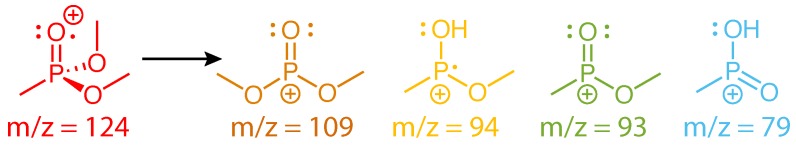
Fragment ions of DMMP${}^{\cdot +}$.

**Scheme 3:**
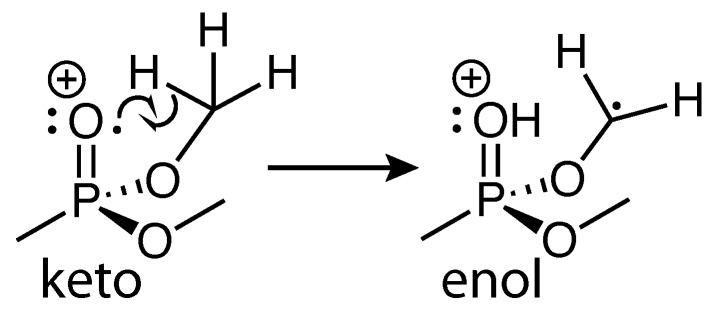
H-atom shift in DMMP${}^{\cdot +}$ to form the enol isomer.

The dissociation products analyzed in Figure 3 have been observed in numerous mass spectrometry studies of DMMP [23,24,25,26,27,28,29,30,31]. Their assigned structures based on these studies and our recently reported theoretical calculations [31] are given in Scheme 2. While the *m*/*z* = 109 and 93 structures result from direct cleavage of a methyl and methoxy group, respectively, the *m*/*z* = 94 and 79 structures involve hydrogen atom migration to the phosphate oxygen atom. This hydrogen migration has been likened to keto-enol isomerization [25] (Scheme 3) and has a transition state barrier of 0.42 eV from the relaxed ion geometry, with only an additional 0.03 eV required to lose CH_2_O and form PO_2_C_2_H_7_^+^ according to our recent calculations [31]. The present FTRMS results indicate that this dissociation pathway can be induced by probe excitation of the parent cation.

#### 2.1.2. DEMP

**Figure 4:**
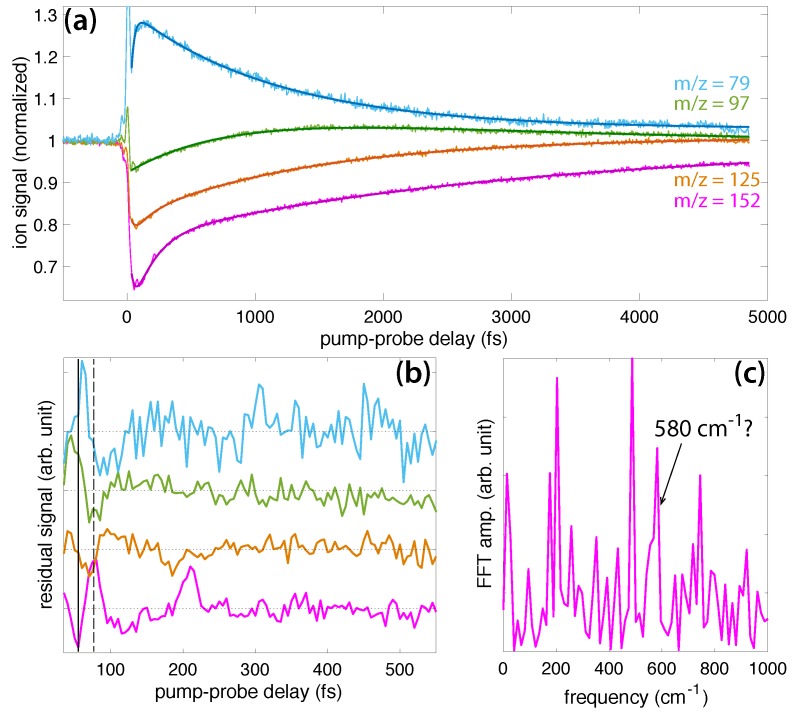
(**a**) Transient signals of ions from DEMP: DEMP${}^{\cdot +}$ (*m*/*z* = 152, magenta), PO_3_H_3_H_10_^+^ (*m*/*z* = 125, orange), PO_3_CH_6_^+^ (*m*/*z* = 97, green), and PO_2_CH_4_^+^ (*m*/*z* = 79, blue). The dark solid lines denote fits to Equation (1). (**b**) Residual ion signals after exponential dynamics are subtracted. (**c**) FFT of residual DEMP${}^{\cdot +}$ signal in (**b**).

Figure 4a shows the transient ion signals of DEMP: DEMP${}^{\cdot +}$ (*m*/*z* = 152, magenta), PO_3_H_3_H_10_^+^ (*m*/*z* = 125, orange), PO_3_CH_6_^+^ (*m*/*z* = 97, green), and PO_2_CH_4_^+^ (*m*/*z* = 79, blue). The signals are normalized to yields at negative time delay as in Figure 3a. The fit coefficients to Equation (1) (solid lines in Figure 4a) are given in the Supplementary Materials (Table S2), and the residual ion signals are plotted in Figure 4b. Unlike in DMMP, the DEMP ion signals exhibit at most one or two visible oscillations, primarily before 100 fs delay, although the parent signal exhibits a revival at 210 fs delay. It is also worth noting that the first minimum (solid line) and maximum (dashed line) of the parent ion signal do not correspond to maxima or minima of the dissociation products, although the PO_3_CH_6_^+^ yield exhibits a minimum at the delay indicated by the dashed line. We hypothesize that this lack of a well-defined phase relationship between the parent and fragment ion oscillations causes extremely rapid disappearance of the oscillations. This situation could arise if the maximum probability of producing each fragment ion occurs at a different position of the vibrational wave packet. The resulting destructive interference between different excitation pathways would preclude clear observation of the wave packet motion in the ion signals. In any case, the lack of well-resolved oscillations precludes effective FFT analysis of the oscillatory dynamics, with no signal clearly visible above the noise (Figure 4c), although the broad width of the feature around 580 cm${}^{-1}$ suggests a possible weak signal.

**Scheme 4:**
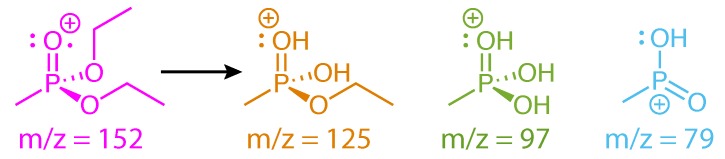
Fragment ions of DEMP${}^{\cdot +}$.

**Scheme 5:**
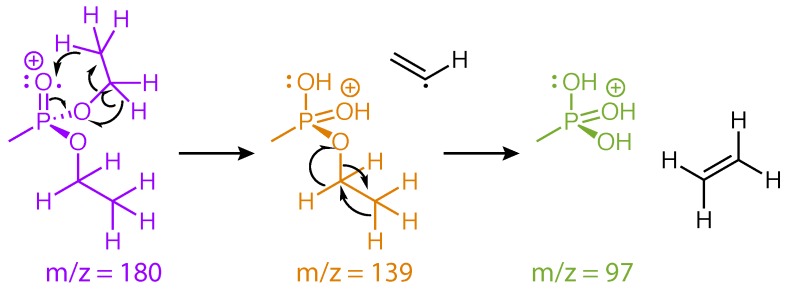
McLafferty rearrangement mechanism in DEMP.

The structures of DEMP${}^{\cdot +}$ and fragment ions taken from the literature [28,29] are shown in Scheme 4. These fragments have been reported to form along a sequential dissociation pathway beginning from the parent ion: *m*/*z* = 152 → 125 → 97 → 79 [29]. The first two steps involve McLafferty rearrangements [32], as shown in Scheme 5. Two hydrogen atoms migrate in the first step, resulting in simultaneous loss of C_2_H_3_ (27 amu) to form the PO_3_C_3_H_10_^+^ ion. A single hydrogen atom migration in the second step results in subsequent loss of C_2_H_4_ (28 amu) to form the PO_3_CH_6_ ion, which can subsequently lose H_2_O to form PO_2_CH_4_^+^ [28,29]. This complex sequence of dissociative rearrangement reactions in DEMP${}^{\cdot +}$ may be expected to destroy any initially prepared vibrational coherence if the hydrogen migrations are sufficiently fast, which would explain the lack of well-defined oscillations in the ion yields shown in Figure 4. However, our results on DIMP do not support this assertion, as discussed below. Therefore, we attribute the lack of long-lived coherence to the phase offsets of each product ion visible in Figure 4b, which will require further investigation of the relevant excited states to explain.

#### 2.1.3. DIMP

**Figure 5:**
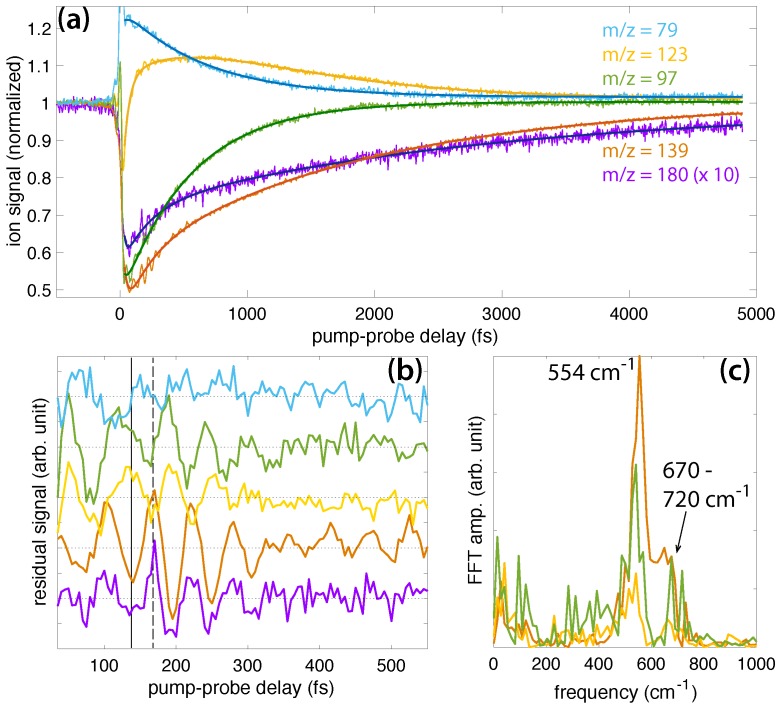
(**a**) Transient signals of ions from DIMP: DIMP${}^{\cdot +}$ (*m*/*z* = 180, violet, magnified by a factor of 10), PO_3_C_4_H_12_^+^ (*m*/*z* = 139, orange), PO_3_C_3_H_8_^+^ (*m*/*z* = 123, gold), PO_3_CH_6_^+^ (*m*/*z* = 97, green), and PO_2_CH_4_^+^ (*m*/*z* = 79, blue). The dark solid lines denote fits to Equation (1). (**b**) Residual ion signals after exponential dynamics subtracted. (**c**) FFT of residual PO_3_C_4_H_12_^+^, PO_3_C_3_H_8_^+^, and PO_3_CH_6_^+^ ion signals in (**b**).

Figure 5a shows transient ion signals of DIMP: DIMP${}^{\cdot +}$ (*m*/*z* = 180, violet), PO_3_C_4_H_12_^+^ (*m*/*z* = 139, orange), PO_3_C_3_H_8_^+^ (*m*/*z* = 123, gold), PO_3_CH_6_^+^ (*m*/*z* = 97, green), and PO_2_CH_4_^+^ (*m*/*z* = 79, blue). Signals are normalized as in Figure 3a and Figure 4a, with the exception that the DIMP${}^{\cdot +}$ signal is magnified by a factor of 10 due to its small yield in the mass spectrum (cf., Figure 1). The coefficients extracted from fitting the ion signals to Equation (1) (dark lines in Figure 5a) are presented in the Supplemental Materials (Table S3). The residual ion signals in Figure 5b show more well-resolved oscillations than DEMP, with the oscillations in PO_3_C_4_H_12_^+^ resolved at delays longer than 500 fs. The solid and dashed lines indicating a minimum and maximum of the DIMP${}^{\cdot +}$ yield, respectively, show that this PO_3_C_4_H_12_^+^ fragment exhibits perfectly in-phase oscillations with the parent ion. In-phase oscillations of parent and fragment ions in polyatomic molecules have not previously been observed in FTRMS experiments [7,8,9,10,11,12,13,14,15,16,17,18,19] and suggest that dissociation of DIMP${}^{\cdot +}$ to form PO_3_C_4_H_12_^+^ occurs spontaneously without probe excitation. The oscillations of the smaller dissociation product PO_3_C_3_H_8_^+^ are approximately antiphase with DIMP${}^{\cdot +}$ and PO_3_C_4_H_12_^+^, suggesting it can be formed upon probe excitation of the parent ion. Meanwhile, the oscillations of PO_3_CH_6_^+^ and PO_3_CH_4_^+^ are somewhat phase-shifted from the larger transients but approximately antiphase with each other. These complex phase relationships may point to the presence of two different wave packets, as suggested in FTRMS experiments on acetophenone [16], although further investigation will be needed to test this hypothesis. FFT of the ion signals over the 30–2500 fs window yields a prominent peak at approximately 554±28 cm${}^{-1}$ and a weaker peak in the range of 670–720 cm${}^{-1}$.

**Scheme 6:**
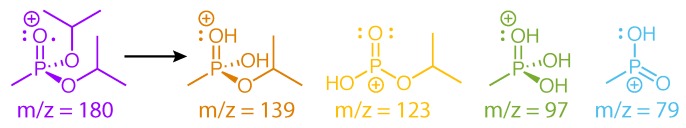
Fragment ions of DIMP${}^{\cdot +}$.

The structures of DIMP fragment ions are shown in Scheme 6. Previous mass spectral studies of DIMP indicate that it undergoes a sequential dissociation pathway analogous to DEMP: *m*/*z* = 180 → 139 → 97 → 79 via two McLafferty rearrangements (Scheme 5) [28,29]. The *m*/*z* = 123 product is formed through the sequence *m*/*z* = 180 → 165 → 123, which involves the direct cleavage of the methyl group followed by C_3_H_6_ loss via a McLafferty rearrangement [29]. While these dissociative rearrangement reactions resemble those in DEMP, the preservation of ion yield oscillations over ∼500 fs in DIMP suggests that these reactions either do not destroy the initial vibrational coherence or occur on a slower timescale than the coherence lifetime.

### 2.2. Assignments of Coherently Excited Vibrational Modes

**Figure 6:**
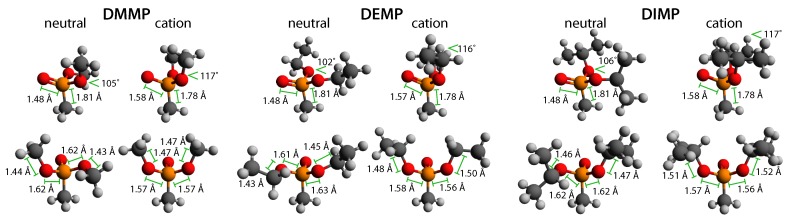
Neutral and cation geometries of DMMP, DEMP, and DIMP with selected bond lengths and angles labeled.

Coherent vibrational excitation in radical cations arises due to the mismatch between the molecular geometries of the neutral and radical cation. For instance, many substituted benzenes exhibit planar or near-planar geometry as neutrals but non-planar geometry as radical cations, which induces coherent excitation of the torsional vibration of the substituent with respect to the benzene ring [11,12,13,14,15,16,17,19]. Our recent results on DMMP indicated that the lengthening of the P=O bond and changing angles of the methoxy substituents induces excitation of the O–P–O bending mode that includes P=O and P–C stretching [18]. To predict whether similar geometrical changes occur in DEMP and DIMP, the neutral and cation geometries were computed at the B3LYP/6-311+G* level. The computed geometries of DMMP agree with previous results [18,31,33] and serve to benchmark the method for DEMP and DIMP. Figure 6 depicts the neutral and cation geometries of DMMP, DEMP, and DIMP with the P=O, P–O, P–C, and C−O bond lengths and O–P–O angle labeled. The geometric coordinates of all atoms in the structures are given in the Supplementary Materials (Tables S4–S6). The associated vertical ionization potentials (IP${}_{\mathrm{vert}}$) and cation relaxation energies (E${}_{\mathrm{relax}}$) for each molecule are listed in Table 1. We note that the location of the unpaired electron in the relaxed cations remained on the oxygen atom initially double-bonded to the phosphorus atom; the known radical migrations in these molecules are associated with hydrogen atom transfers shown in Scheme 3 and Scheme 5 [24,25,26,27,28,29,30,31,32].

Ionization induces a number of analogous geometrical changes across the methylphosphonate series, as observed in Figure 6. First, the P=O bond lengthens by 6.8% and the P–C bond shortens by 1.7% in each molecule. Second, the O–P–O angle increases from 102–106${}^{\circ }$ in the neutrals to $117^{\circ }$ in the cations. Third, P–O single bonds shorten by 1.9–4.3% in each molecule, but DMMP${}^{\cdot +}$ exhibits identical P–O bond lengths, while one of the P–O bonds becomes slightly shorter than the other in both DEMP${}^{\cdot +}$ and DIMP${}^{\cdot +}$. Finally, both C–O bonds lengthen by approximately 3% in each cation. These geometric changes are expected to induce coherent excitation along one or more associated vibrational modes in each molecule.

**Figure 7:**
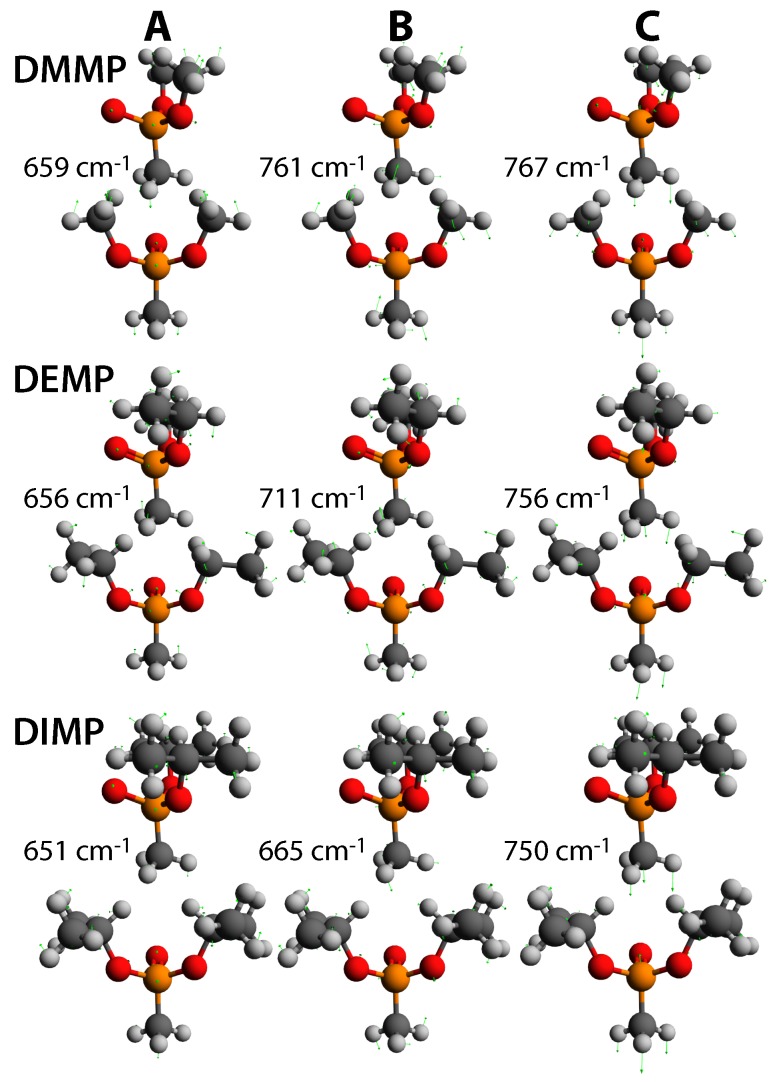
Potential coherently excited vibrational modes in DMMP, DEMP, and DIMP.

To identify potential coherently excited vibrations, the normal modes for both the neutral and cation geometries of DMMP, DEMP, and DIMP were computed and compared to experimental IR and Raman measurements [34,35,36,37] (Supplementary Materials, Tables S7–S9 and Figures S1–S3). The rough agreement of the computed spectra of the DMMP, DEMP, and DIMP neutrals with experimental measurements is similar to recent computational studies of the IR spectra of these molecules [33,38]. Based on these computed normal modes, the ionization-induced geometrical changes, and experimentally observed oscillation frequencies, we focused on a cluster of three normal modes labeled **A**, **B**, and **C** with computed frequencies for the neutrals and cations given in Table 2 and illustrations of the motions given in Figure 7. Modes **A** and **B** have been defined as symmetric and asymmetric O–P–O bending modes, respectively, and Mode **C** as the P–C stretch [39], although additional motions are clear in Figure 7.

The 732±28 cm${}^{-1}$ coherent oscillations in DMMP${}^{\cdot +}$ were previously assigned to Mode **C** [18]. This mode was computed at 752 cm${}^{-1}$ using an anharmonic correction [18], and our current computations with no anharmonic correction predict this mode at 767 cm${}^{-1}$, in agreement with the previous uncorrected results in Ref. [18] (Supplementary Materials, Table S7). The additional shoulder peak at 610–650 cm${}^{-1}$ visible in Figure 3c can be attributed to Mode **A**, the symmetric O–P–O bend. While Modes **B** and **C** have almost identical frequencies, excitation of Mode **C** is more likely because it involves symmetric motions of the two methoxy substituents, which have nearly identical geometric parameters in the DMMP${}^{\cdot +}$ (Figure 6). As a result, it is unlikely that the asymmetrical motions of Mode **B** would be excited by ionization.

In DEMP, the lack of sufficient resolution of oscillations precludes the assignment of any coherently excited normal modes, although the potential weak feature around 580 cm${}^{-1}$ in Figure 4c could arise from excitation of Mode **A**. In DIMP, the strong feature at 554±28 cm${}^{-1}$ observed in Figure 5c is most likely due to Mode **A**, even though the calculated frequency exceeds the experimental frequency by around 100 cm${}^{-1}$ because the geometrical changes in P–O and C–O bond lengths in Figure 6 are consistent with the motions of mode **A** in Figure 7. The weak peak in the range of 670–720 cm${}^{-1}$ is most likely attributable to mode **C**; a similar overestimation of this frequency in the calculations is observed. As with DMMP, excitation of Mode **B** in DIMP${}^{\cdot +}$ is unlikely due to its asymmetric motion and the symmetric geometrical changes upon ionization.

## 3. Discussion

The FTRMS measurements presented here demonstrate how the coherent vibrational motions of polyatomic radical cations may be investigated with only mass spectrometric detection. With the assistance of DFT calculations, the coherent excitation of the same two vibrational modes in DMMP${}^{\cdot +}$ and DIMP${}^{\cdot +}$ could be identified. While no definitive mode assignments could be made in DEMP${}^{\cdot +}$ due to the relative lack of visible coherent dynamics, the excitation of one of the same modes is suggested in the data. These results add to the growing body of FTRMS studies of coherent vibrational dynamics in polyatomic radical cations [7,8,9,10,11,12,13,14,15,16,17,18,19]. The observed coherent dynamics in these studies are assumed to arise from vibrational motion on the electronic ground state of the cation, which requires its selective population during the ionization process. Strong-field excitation at the 1500 nm wavelength used in this work is known to produce greater cationic ground state population than the Ti:Sapphire wavelength of 800 nm due to a well-established adiabatic electron tunneling ionization mechanism [15,18,40]. Moreover, the strong electric fields required for non-resonant femtosecond laser ionization typically remove an electron before significant neutral excited state population can occur [14]. However, strong-field tunneling ionization still populates ionic excited states in the organic phosphonates, as evident in the large yields of small fragment ions in Figure 1. This limitation of FTRMS may ultimately be overcome upon the development of facile methods of producing femtosecond pulses in the VUV spectral range, which would allow for single-photon ionization.

This result that ionization induces coherent excitation of the same vibrational modes across the methylphosphonate series points to a generic vibrational excitation mechanism in ionized organic phosphonates. Additionally, the similar cation relaxation and dissociation dynamics of each molecule reflected in the observed exponential decay signals suggests common mechanistic pathways across the series. These results, combined with the well-known shared intramolecular rearrangement and dissociation pathways of organic phosphonates with the related phosphates [23,24,28,29,32], raise the possibility that analogous vibrational excitation and dissociation mechanisms operate across a broad class of biologically relevant organophophorous compounds. Further investigation of these dynamical pathways may lead to enhanced understanding of the mechanisms leading to radiation-induced DNA damage. For instance, these pathways may explain the observed rapid formation of sugar-centered radicals in one-electron oxidized DNA [2]. The presence of common vibrational excitations and dissociation dynamics in the radical cations of methylphosphonates also mirrors shared vibrational excitations in classes of organic radical cations including alkyl phenyl ketones [13,14], nitrotoluenes [17,19], and halogenated methanes [7].

**Figure 8:**
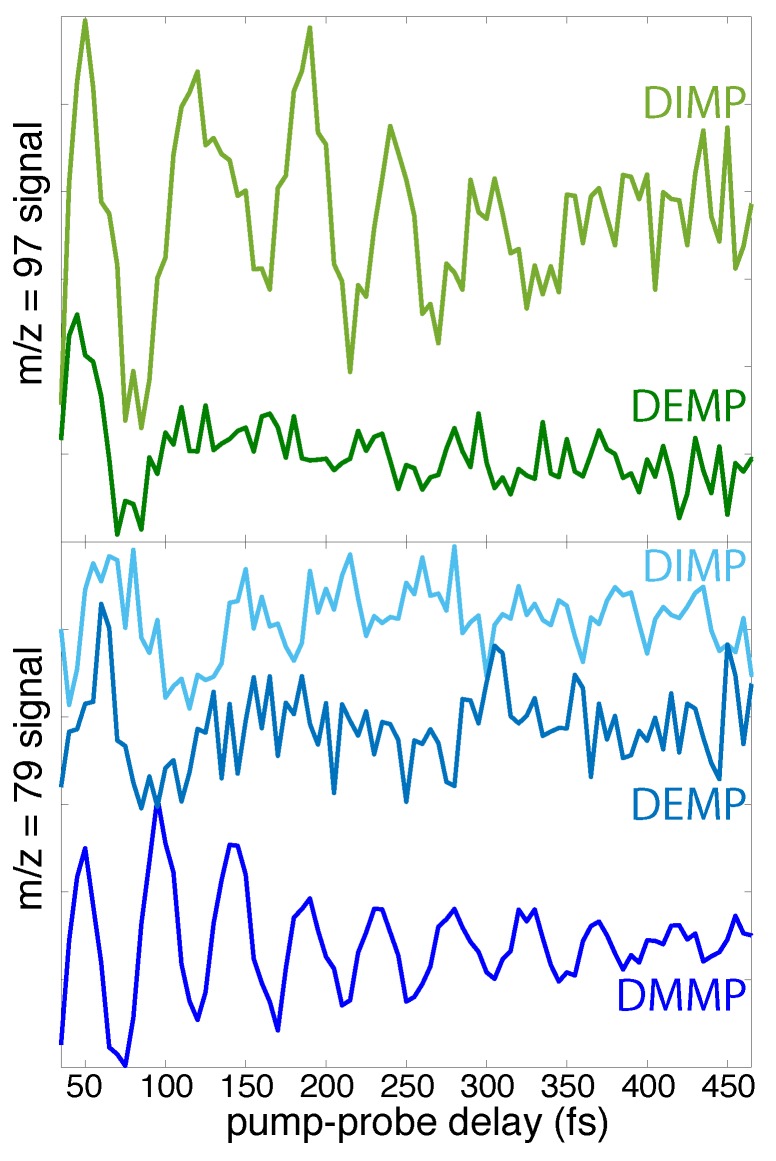
Oscillatory components of *m*/*z* = 97 (**top**) and *m*/*z* = 79 (**bottom**) signals in DMMP, DEMP, and DIMP.

In addition to the common vibrational excitation and dissociation pathways observed across the methylphosphonate series, the distinct oscillation periods and dynamics observed in each molecule suggest that FTRMS may enable discrimination among possible source molecules in a complex mixture that give rise to mass spectral peaks with the same *m*/*z* value. As an illustration, the residual oscillatory dynamics of the peaks at *m*/*z* = 97 and *m*/*z* = 79 (Figure 8) are highly dependent on the source molecule. Both the oscillation period and duration of observable oscillations are different in DMMP, DEMP, and DIMP. Despite the noisy signals in the present data that preclude resolution in a mixture, significant improvements in signal resolution could, in principle, enable the deconvolution of multiple oscillatory signals by FFT or other methods. In particular, further refinement of the FTRMS technique single-photon VUV ionization and tunable probe excitation wavelengths may provide the necessary signal resolution. This potential ability of FTRMS to obtain vibrational information from mass spectrometry measurements could significantly enhance the capabilities of modern analytical mass spectrometry techniques such as femtosecond laser desorption-postionization, which has been used to image biological materials including microbial biofilms and mammalian tissue [41,42,43,44,45]. In particular, replacing the commonly used VUV ionization laser with a pump–probe pulse pair could provide a coherent vibrational “fingerprint” of constituent molecules detected in the mass spectrometer. This combination of FTRMS with desorption techniques therefore has the potential to yield new detection and discrimination capabilities for analysis of biological samples.

## 4. Materials and Methods

### 4.1. Materials

DMMP (97%) and DEMP (97%) were purchased from Sigma-Aldrich (St. Louis, MO, USA), and DIMP (95%) was purchased from Alfa Aesar (Tewksbury, MA, USA). All samples were used as received. A small toluene impurity in the DIMP sample was observable in the mass spectrum upon initial introduction, but disappeared after a few days; all DIMP data were recorded after the toluene disappearance.

### 4.2. Experimental Methods

The experimental setup has been described in detail in our previous publications [17,18,31,46]; a brief summary is given here. The output of a commercial Ti:Sapphire regenerated amplifier (Astrella, Coherent, Inc., Santa Clara, CA, USA) producing 30 fs, 800 nm, 2.2 mJ pulses was split with a 10:90 (r:t) beam splitter. The 90% portion was directed into an optical parametric amplifier (OPA, TOPAS Prime) to produce 1500 nm, 200 μJ, 18 fs pulses used as the pump. The smaller portion of the beam used as the probe pulse was directed onto a retro-reflector placed on a motorized stage to adjust the delay between pump and probe pulses. With a home-built frequency resolved optical gating (FROG) apparatus [47], the duration of the pump and probe pulses were measured to be 18 fs and 25 fs, respectively. The pump and probe beams are recombined on a dichroic mirror and focused with a 20 cm fused silica biconvex lens to into the extraction region of a custom built time-of-flight (TOF) mass spectrometer. The focal intensities of the pump and probe pulses were estimated as $1.5\times 10^{14}$ and $1.5\times 10^{13}$ W cm${}^{-2}$, respectively, based on previously established methods described in Ref. [46]. The molecular sample was introduced into the ultrahigh vacuum chamber (base pressure $2\times 10^{-9}$ Torr) through the effusive source of a 1/16” OD stainless steel tube with outlet 1 cm away from the laser focus. The resulting sample pressure was kept at approximately $2\times 10^{-7}$ Torr as measured near the microchannel plate detector. Mass spectra at each pump–probe delay were collected by a 1 GHz digital oscilloscope (LeCroy WaveRunner, Chestnut Ridge, NY, USA) and averaged over 1000 laser shorts. The pump–probe delay was scanned in steps of 5 fs over the approximate range from −500 fs to +5000 fs, and 40 scans were averaged to produce the reported experimental data.

### 4.3. Theoretical Methods

Our density functional theory (DFT) calculations to interpret the coherent vibrational dynamics of the DMMP, DIMP, and DEMP cations were conducted using Gaussian 16 suite of programs [48]. All computations were performed using Gaussian 6-311+G* (5s4p1d) basis set [49] and generalized for all atoms. Neutral geometries were optimized beginning from geometries obtained from the PubChem database [50]. Chemcraft software [51] was used to specify $C_{s}$ symmetry for the starting geometry of each cation optimization based on the previous finding of nearly $C_{s}$ symmetry for DMMP${}^{\cdot +}$ [18,31]. The optimized geometries and vibrational spectra of each neutral and cation were evaluated using the generalized approximation (DFT-GGA) and hybrid Hartree–Fock method B3LYP [52]. These methods produced energies of DMMP and DMMP${}^{\cdot +}$ within 0.01 eV of previous literature [18,31]. The same methods were applied to compute optimized geometries and vibrational frequencies of DIMP, DIMP${}^{\cdot +}$, DEMP, and DEMP${}^{\cdot +}$. The vertical ionization potentials and relaxation energies for all species were evaluated with the same methods.

## 5. Conclusions

Femtosecond time-resolved mass spectrometry was used to record coherent vibrational dynamics in radical cations of the methylphosphonate series of molecules DMMP, DEMP, and DIMP. The time-domain measurements recorded oscillations in ion yields as a function of pump–probe delay extending to ∼500 fs after ionization with a period of 45 fs in DMMP and 55 fs in DIMP. FFT analysis of the oscillatory ion signals yielded a strong 732±28 cm${}^{-1}$ peak and weak 610–650 cm${}^{-1}$ feature in DMMP and strong and weak peaks at 554±28 cm${}^{-1}$ and 670–720 cm${}^{-1}$, respectively, in DIMP. DEMP exhibited less well-defined oscillations in ion yields and no definitive frequency retrievable with FFT, although a weak feature around 580 cm${}^{-1}$ barely visible above the noise was observed. DFT calculations of the cation vibrational modes in the cations enabled assignment of the low- and high-frequency features in DMMP and DIMP to the symmetric O–P–O bend and P–C stretch modes, respectively, and possible assignment of the weak DEMP oscillations to the O–P–O bend. These results demonstrate the ability of FTRMS measurements to directly extract information about vibrational excitations in polyatomic cations that are models for biologically relevant organophosphorus compounds using only mass spectrometric detection and can inform future development of the FTRMS technique for bioanalytical applications.

## Figures and Tables

**Table 1:** Computed ionization and relaxation energies.

Molecule	Neutral E (Hartree)	Relaxed Cation E (Hartree)	IP_vert_ (eV)	E_relax (eV)_
DMMP	−686.8138	−686.4663	10.31	0.66
DEMP	−765.4112	−765.0739	9.82	0.64
DIMP	−844.0129	−843.6786	9.55	0.58

**Table 2:** Computed vibrational energies of DMMP, DEMP, and DIMP in cm${}^{-1}$. Experimental values given in parentheses. *a*: Ref. [36]; *b*: Ref. [34]; *c*: Ref. [35].

Mode	DMMP Neutral	Cation	DEMP Neutral	Cation	DIMP Neutral	Cation
**A**	672 (712${}^{a}$)	659	689 (715${}^{b}$)	656	690 (719${}^{c}$)	651
**B**	759 (786${}^{a}$)	761	765 (771${}^{b}$)	711	742 (748${}^{c}$)	665
**C**	794 (818${}^{a}$)	767	788 (806${}^{b}$)	756	778 (791${}^{c}$)	750

## Supplementary Materials

The following are available online, Tables S1–S10; Figures S1–S3.

## Abbreviations

The following abbreviations are used in this manuscript:

FTRMS	Femtosecond time-resolved mass spectrometry
DNA	Deoxyribonucleic acid
DMMP	Dimethyl methylphosphonate
DEMP	Diethyl methylphosphonate
DIMP	Diisopropyl methylphosphonate
FFT	Fast Fourier transform
