# Supplementary material for: Probing Coherent Vibrations of Organic Phosphonate Radical Cations with Femtosecond Time-Resolved Mass Spectrometry
Source: Molecules. 2019 Jan 31;24(3):509. doi: 10.3390/molecules24030509 (PMC6384684; doi:10.3390/molecules24030509)
Supplement: Supplementary file 1 [file molecules-24-00509-s001.pdf]

Supplementary Materials: Probing Coherent Vibrational Motions of Organic Phosphonate Radical Cations with Femtosecond Time-Resolved Mass Spectrometry

Derrick Ampadu Boateng, Mi'Kayla D. Word, and Katharine Moore Tibbetts *

1. Tabulated dynamical fit coefficients

All transients were fit to the equation

$$S(\tau) = a \exp\left(-\tau/T_1\right) + b \exp\left(-\tau/T_2\right) + c \exp\left(-\tau/T_3\right) + d. \tag{1}$$

Some transients only required two exponential decays for fitting.

Table S1. Fit coefficients for ions associated with DMMP.

species	m/z	a	T_1 (fs)	b	T_2 (fs)	c	T_3 (fs)	d
$DMMP^{\cdot+}$	124	0.15 ± 0.09	37 ± 34	-0.18 ± 0.12	112 ± 54	-0.042 ± 0.019	541 ± 153	0.849 ± 0.001
$PO_3(CH_3)_2^+$	109	-	-	0.029 ± 0.027	299 ± 143	-0.056 ± 0.029	629 ± 116	0.412 ± 0.001
$PO_2C_2H_7^+$	94	0.66 ± 0.21	16 ± 12	-0.050 ± 0.008	152 ± 28	-0.14 ± 0.02	7900 ± 2800	0.963 ± 0.002
$PO_2(CH_3)_2^+$	93	-0.17 ± 0.02	30 ± 8	0.054 ± 0.030	91 ± 23	0.051 ± 0.002	2820 ± 160	0.400 ± 0.002
$PO_2CH_4^+$	79	-	-	0.079 ± 0.022	190 ± 47	-0.092 ± 0.025	538 ± 69	1.051 ± 0.001

Table S2. Fit coefficients for ions associated with DEMP.

species	m/z	a	T_1 (fs)	b	T_2 (fs)	c	T_3 (fs)	d
$DEMP^{\cdot+}$	152	0.31 ± 0.05	30 ± 5	-0.224 ± 0.023	168 ± 9	-0.229 ± 0.002	3020 ± 120	0.993 ± 0.004
$PO_3C_3H_{10}^+$	125	0.15 ± 0.05	28 ± 12	-0.067 ± 0.025	122 ± 33	-0.194 ± 0.002	1390 ± 30	0.885 ± 0.001
$PO_3CH_6^+$	97	-	-	-0.22 ± 0.13	850 ± 190	0.15 ± 0.13	1900 ± 900	0.894 ± 0.007
$PO_2CH_4^+$	79	-0.51 ± 0.11	26 ± 3	-	-	0.285 ± 0.003	1150 ± 30	2.430 ± 0.001

Table S3. Fit coefficients for ions associated with DIMP.

species	m/z	a	T_1 (fs)	b	T_2 (fs)	c	T_3 (fs)	d
$DIMP^{\cdot+}$ ($\times$10)	180	0.21 ± 0.19	27 ± 22	-0.16 ± 0.03	202 ± 45	-0.27 ± 0.01	2740 ± 290	0.987 ± 0.012
$PO_3C_4H_{12}^+$	139	0.80 ± 0.13	30 ± 6	-0.35 ± 0.03	203 ± 21	-0.86 ± 0.01	1880 ± 60	2.185 ± 0.004
$PO_3C_3H_8^+$	123	-0.88 ± 0.05	43 ± 4	-0.84 ± 0.51	570 ± 120	0.95 ± 0.53	990 ± 160	2.804 ± 0.003
$PO_3CH_6^+$	97	0.05 ± 0.03	27 ± 11	-0.20 ± 0.01	548 ± 5	-	-	1.001 ± 0.001
$PO_2CH_4^+$	79	-0.02 ± 0.01	48 ± 24	0.11 ± 0.01	659 ± 12	-	-	1.007 ± 0.001

2. Optimized Geometries

	Neutral			Cation		
Element	x	y	z	x	y	z
P	-0.014798	0.499741	0.174628	-0.000054	0.507481	0.083585
O	-0.964243	-0.411597	-0.778099	-0.000174	0.320753	1.654961
O	-0.710834	1.225992	1.257092	-0.000293	2.181018	-0.50882
O	1.08155	-0.531014	0.780944	-0.000293	2.1878	-1.600081
C	-2.130662	-1.058085	-0.233213	-0.892614	2.68898	-0.138944
C	0.84338	1.505777	-1.056917	0.891867	2.689262	-0.138945
C	1.815132	-1.468547	-0.016988	1.334372	-0.148877	-0.41047
H	-1.842344	-1.867369	0.441681	-1.334314	-0.149246	-0.410504
H	-2.748836	-0.338469	0.304087	1.909402	-1.481877	-0.179937
H	-2.676687	-1.464684	-1.082457	1.499939	-1.9225	0.726636
H	1.282463	0.894782	-1.846899	1.684189	-2.083067	-1.058767
H	0.129842	2.198884	-1.503579	2.978904	-1.32328	-0.085238
H	1.62781	2.078782	-0.560029	-1.908927	-1.482438	-0.180021
H	1.150056	-2.030548	-0.676427	-1.683859	-2.083377	-1.05906
H	2.579006	-0.959497	-0.611399	-1.499035	-1.923143	0.726317
H	2.30177	-2.149918	0.678811	-2.978439	-1.324143	-0.084918

Table S4. Geometric coordinates for DMMP and $DMMP^{\cdot +}$

	Neutral			Cation		
Element	x	y	z	x	y	z
P	-0.022449	-0.860151	0.194661	-0.03881	0.769557	0.067858
O	1.38272	-0.107128	0.430131	1.335906	0.164358	-0.351514
O	-0.50713	-1.567862	1.399328	-1.311939	0.18232	-0.662125
O	-0.974237	0.348067	-0.331251	-0.347882	0.40432	1.567596
C	3.178925	1.463471	0.224519	1.916702	-1.198381	-0.14098
C	1.935461	0.907476	-0.438178	-1.908783	-1.176146	-0.629734
C	0.218942	-1.918157	-1.257594	0.021392	2.502551	-0.323454
C	-2.377147	0.426661	0.026239	3.396458	-1.044255	0.094692
C	-2.826268	1.865924	-0.116637	-3.073997	-1.207402	0.329998
H	3.919434	0.677758	0.388537	1.69084	-1.742316	-1.05821
H	3.627797	2.233223	-0.410102	1.39898	-1.654265	0.703418
H	2.934863	1.911452	1.189867	-1.126367	-1.893511	-0.381458
H	2.176669	0.456565	-1.407384	-2.219335	-1.321162	-1.66552
H	1.182885	1.682835	-0.597168	0.840815	2.965376	0.229384
H	0.566348	-1.346503	-2.12004	0.188644	2.633856	-1.393795
H	0.948924	-2.69276	-1.017128	-0.923633	2.965797	-0.036913
H	-0.726986	-2.399479	-1.512246	3.88239	-0.547635	-0.746157
H	-2.502883	0.061056	1.046745	3.600728	-0.485295	1.00921
H	-2.940073	-0.229729	-0.645384	3.837448	-2.039137	0.200741
H	-2.27077	2.516841	0.562195	-2.737993	-1.043539	1.357758
H	-2.679661	2.225392	-1.137903	-3.822337	-0.456602	0.07293
H	-3.890119	1.948741	0.12433	-3.547348	-2.191099	0.275947

Table S5. Geometric coordinates for DEMP and $DEMP^{\cdot +}$

	Neutral			Cation		
Element	x	y	z	x	y	z
P	-0.047796	1.047264	-0.205076	0.016549	1.035087	-0.148835
O	1.257769	0.210018	-0.669302	-1.34235	0.295558	-0.429419
O	-0.671371	1.727078	-1.36267	-0.018879	1.309494	1.402404
O	-0.989666	-0.013013	0.583312	1.325369	0.239822	-0.458487
C	3.370265	-0.159369	0.447494	-2.623375	-1.678717	-0.919996
C	2.062828	-2.049648	-0.625069	-2.941775	-0.408444	1.273431
C	1.99479	-0.741698	0.149953	-1.977606	-0.911604	0.218752
C	0.47548	2.116208	1.157475	0.031164	2.475582	-1.194905
C	-3.329225	0.008263	-0.095129	3.136829	-0.349566	1.06126
C	-2.028836	-0.781589	-0.106885	1.988124	-0.921345	0.25849
C	-2.127138	-2.117374	0.611476	2.38972	-1.893895	-0.831313
H	3.295757	0.779913	1.000066	2.784123	0.354305	1.81836
H	3.959916	-0.858387	1.047632	3.652817	-1.164551	1.574831
H	3.910432	0.035589	-0.482291	3.857888	0.153254	0.413879
H	2.616063	-2.803967	-0.058165	-1.890299	-1.982683	-1.668389
H	1.062383	-2.438098	-0.826085	-3.089474	-2.579905	-0.513883
H	2.567336	-1.898694	-1.582623	-3.398857	-1.082866	-1.404205
H	1.443132	-0.901161	1.082451	-3.435128	-1.263012	1.742561
H	0.900315	1.541725	1.982225	-2.417968	0.151926	2.051947
H	1.218239	2.823401	0.785436	-3.71121	0.228166	0.832522
H	-0.388725	2.67177	1.523527	-1.164167	-1.485136	0.667471
H	-3.206148	0.95824	-0.61651	0.043345	2.172663	-2.242995
H	-4.119225	-0.558202	-0.597224	-0.860658	3.071392	-0.996311
H	-3.649826	0.205064	0.931959	0.923218	3.065414	-0.978277
H	-1.70594	-0.936073	-1.141053	1.228223	-1.350586	0.914302
H	-1.175038	-2.651937	0.585093	1.526966	-2.243669	-1.400538
H	-2.409674	-1.972267	1.657614	3.105305	-1.439237	-1.518568
H	-2.884895	-2.747309	0.137482	2.866021	-2.762834	-0.37047

Table S6. Geometric coordinates for DIMP and DIMP$^{\cdot+}$

3. Vibrational Frequencies

Mode	Expt. (cm -1)	Neutral (cm-1)	Int. (km/mol)	Ion ref. (cm -1)	Int. (km/mol)	Ion (cm -1)	Int. (km/mol)
		71.08	1.215	19.27	0.00	12.43	0.001
		85.4	0.704	69.54	0.23	68.83	0.037
		97.11	0.023	84.03	2.96	84.12	0.787
		128.24	0.635	99.72	2.46	98.32	0.686
		178.08	0.383	133.60	4.54	133.85	1.280
		189.38	3.479	154.69	0.77	159.95	0.135
		204.26	0.924	166.52	15.15	166.40	4.086
		276.23	0.085	204.71	10.28	204.37	2.796
		287.26	3.677	253.09	3.46	253.22	0.924
CPO3 wag	398	397.87	9.165	271.82	8.01	271.93	2.162
O2P=O bend	468	433.09	4.433	346.10	10.01	346.17	2.699
CPO3 wag	500	482.78	9.752	471.50	26.80	471.49	7.181
O-P-O bend	712	672.73	2.711	659.12	24.98	659.07	6.710
O-P-O bend	786	759.26	40.796	760.83	0.64	760.81	0.168
P-C str	818	793.6	36.127	766.97	4.70	766.84	1.255
P-CH3 rock	894	922.86	7.419	864.02	13.13	863.96	3.517
P-CH3 rock	912	937.99	35.145	934.66	15.19	935.34	4.086
COPOC str	1037	1056.41	100.000	952.94	34.93	954.07	9.448
COPOC str	1064	1076.41	64.606	1044.26	180.21	1043.92	48.492
O-CH3 rock	1152	1181.6	0.262	1065.54	371.47	1065.01	100.000
O-CH3 rock	1152	1184.52	0.439	1165.06	2.74	1164.95	0.680
O-C stretch	1183	1202.9	4.157	1168.16	0.20	1167.98	0.056
O-C stretch	1183	1203.46	5.640	1193.50	31.98	1193.56	8.904
P=O str	1250	1275.09	76.116	1193.89	17.05	1193.72	4.732
PC-H bend	1380	1360.66	12.204	1375.94	32.59	1374.53	8.331
PC-H bend	1418	1472.61	1.575	1454.26	17.40	1455.65	4.511
PC-H bend	1418	1479.49	1.616	1454.42	10.49	1456.31	2.559
H-C-H bend		1485.92	0.389	1476.73	3.58	1476.11	1.226
H-C-H bend		1489.49	0.356	1478.22	4.47	1477.63	1.178
H-C-H bend	1450	1507.92	2.742	1481.76	13.10	1481.81	3.263
H-C-H bend	1463	1515.3	1.698	1491.56	0.64	1491.50	0.176
H-C-H bend	1463	1518.03	4.173	1502.53	7.20	1502.83	1.595
H-C-H bend	1463	1521.15	2.048	1507.95	59.09	1507.91	15.692
OC-H3 str	2848	3031.4	15.030	3059.30	16.93	3059.09	4.576
OC-H3 str	2848	3044.28	14.677	3078.34	0.53	3078.18	0.144
PC-H3 str	2848	3055.89	0.757	3079.80	7.36	3079.65	1.996
OC-H3 str	2951	3100.5	9.906	3149.72	6.74	3149.84	1.777
OC-H3 str	2951	3120.45	7.639	3150.15	5.39	3150.13	1.413
OC-H3 str	2992	3137.23	1.777	3176.98	2.61	3176.84	0.690
OC-H3 str	2992	3140.57	6.394	3177.53	2.66	3177.33	0.664
PC-H3 str	2951	3141.44	0.301	3201.86	1.45	3202.01	0.386
PC-H3 str	2951	3150.11	5.003	3202.33	0.65	3202.51	0.181

Table S7. Vibrational frequencies for DMMP and $DMMP^{\cdot+}$ with associated mode intensities. Experimental reference [1,2]; computational ion reference [3].

Mode	Expt. (cm -1)	Neutral (cm -1)	Int. (km/mol)	Ion (cm -1)	Int. (km/mol)
		16.30	0.333	29.77	0.015
		28.30	0.355	44.66	0.009
		43.62	0.497	60.96	0.311
		69.42	1.088	70.78	0.169
		116.09	0.705	138.92	1.866
		171.88	0.673	147.04	1.375
		192.73	0.479	155.17	0.707
		240.95	0.187	211.98	0.336
		247.68	0.030	241.67	0.498
		263.02	0.713	250.34	0.700
		279.85	0.563	259.06	0.192
		323.72	1.956	263.22	1.245
		332.74	2.139	292.09	2.228
		417.28	4.145	378.42	0.686
CPO3 wag	485	444.55	11.828	440.27	2.766
O-P-O bend	502	480.25	5.021	467.91	5.268
O-P-O bend	715	689.28	1.704	655.65	7.359
P-C str	771	764.67	22.354	709.84	1.504
	806	787.93	28.244	755.85	1.541
		818.99	0.246	813.97	0.657
		821.16	0.077	816.16	1.711
P-CH3 rock	898	917.17	3.836	821.73	0.699
P-CH3 rock	939	927.42	7.797	926.33	4.193
C-C str	965	954.61	50.456	946.71	5.419
	965	968.19	48.592	967.02	6.268
COPOC str	1023	1053.05	100.000	983.21	7.839
COPOC str	1049	1073.88	50.812	1004.12	54.956
O-C str	1099	1123.98	2.263	1034.33	100.000
		1127.89	2.410	1106.47	2.537
CH3 rock	1164	1182.82	0.889	1122.68	2.319
		1185.53	1.120	1165.09	3.104
P=O str	1250	1271.33	68.912	1172.72	1.523
PC-H bend	1314	1318.29	0.060	1305.31	4.221
		1321.01	0.590	1315.02	1.594
		1357.16	9.654	1372.59	7.367
PC-H bend	1390	1409.94	0.377	1386.53	2.133
PC-H bend	1390	1411.10	1.014	1403.12	2.509
H-C-H bend	1420	1433.10	3.940	1420.28	3.704
H-C-H bend	1420	1434.48	5.134	1434.31	4.638
H-C-H bend	1445	1476.03	1.433	1456.83	4.466
H-C-H bend	1480	1481.53	0.966	1459.37	2.494
H-C-H bend	1480	1499.52	1.905	1480.58	1.076
		1500.55	2.311	1488.39	8.464
		1514.16	0.591	1496.04	3.533
		1514.58	1.128	1504.08	0.171
		1535.46	0.792	1510.66	1.705
		1537.12	0.701	1516.83	1.613
CC-H3 str	2863	3025.10	9.404	3037.83	2.609
CC-H3 str	2863	3034.68	10.899	3053.10	0.257
CC-H3 str	2935	3037.73	6.114	3059.91	3.375
CC-H3 str	2935	3040.07	5.129	3078.31	6.861
CC-H3 str	2943	3049.68	1.068	3081.57	1.591
CC-H3 str	2943	3085.45	1.859	3107.71	5.158
C-H2 str	2885	3093.18	0.420	3120.34	1.536
C-H2 str	2885	3103.15	8.794	3126.10	1.180
C-H2 str	2990	3105.26	8.358	3127.48	0.475
C-H2 str	2990	3118.75	9.659	3145.94	2.353
PC-H3 str	2880	3120.76	10.167	3147.30	1.054
PC-H3 str	2990	3129.02	2.329	3151.83	1.923
PC-H3 str	2995	3134.18	1.272	3153.95	0.862

Table S8. Vibrational frequencies for DEMP and $DEMP^{\cdot+}$ with associated mode intensities. Experimental reference [2,4].

Mode	Expt. (cm -1)	Neutral (cm -1)	Int. (km/mol)	Ion (cm -1)	Int. (km/mol)
		26.32	0.250	8.90	0.007
		36.26	0.071	24.76	0.001
		53.42	0.032	46.36	0.048
		67.34	0.383	57.49	0.188
		134.11	1.049	105.58	1.360
		140.55	1.241	109.14	0.458
		192.35	0.008	151.44	0.201
		220.60	0.021	161.18	2.273
		221.56	0.058	224.53	0.401
		227.79	0.013	225.03	0.310
		249.05	0.150	226.83	0.462
		256.91	0.707	232.22	0.292
		265.53	0.402	240.44	0.834
		292.69	1.261	268.65	0.132
		346.53	0.252	276.73	0.349
		353.25	0.147	334.40	0.233
		389.72	1.488	356.82	0.671
		415.06	4.963	403.40	0.514
		438.94	2.615	409.51	4.596
	451	462.55	1.742	450.41	0.899
CPO3 wag	504	490.06	7.639	461.96	1.763
O2P=O bend	541	500.48	5.912	465.85	4.488
O-P-O bend	719	690.02	2.159	650.80	13.890
O-P-O bend	748	741.63	8.579	664.81	5.215
P-C str	791	777.52	17.159	749.86	1.229
C-C-C bend	884	880.12	4.982	810.53	1.018
C-C-C bend	884	882.73	8.083	873.89	8.169
P-CH3 rock	899	922.14	7.532	884.06	5.642
P-CH3 rock	917	936.08	32.931	930.01	4.038
C-H bend	938	941.32	3.346	946.16	0.312
C-H bend	938	943.90	1.036	946.91	0.142
		948.99	0.151	949.88	0.273
		952.49	0.185	951.48	0.232
COPOC str	994	973.35	100.000	952.18	0.111
COPOC str	1018	1002.87	68.420	969.67	53.153
O-C3 str	1110	1134.15	7.995	1001.41	100.000
O-C3 str	1115	1135.58	11.181	1097.60	3.849
C-C str	1115	1157.35	2.393	1103.01	3.943
C-C str	1115	1161.69	2.923	1147.50	3.343
C-C str	1177	1204.26	6.260	1156.33	2.151
C-C str	1183	1206.19	2.781	1192.30	2.080
P=O str	1251	1266.82	54.292	1200.19	1.130
C-H bend	1350	1358.40	8.470	1357.22	3.329
PC-H bend	1362	1371.81	0.551	1362.71	14.660
		1372.98	0.883	1364.76	2.867
C-H bend	1385	1398.45	2.779	1372.15	9.333
C-H bend	1390	1399.31	3.394	1376.16	1.442
PC-H bend	1419	1415.64	2.578	1414.62	5.234
PC-H bend	1419	1418.06	4.590	1420.01	6.141
		1431.61	7.521	1430.76	0.534
		1432.66	2.372	1435.13	0.516
H-C-H bend	1453	1474.50	1.259	1459.17	5.255
H-C-H bend	1453	1482.20	1.194	1460.76	2.899
H-C-H bend	1475	1496.22	0.375	1484.44	2.091
H-C-H bend	1475	1496.64	0.377	1489.20	1.329
		1501.61	0.118	1491.95	0.760
		1502.79	0.221	1493.67	0.576
		1510.44	1.795	1499.86	10.238
		1512.09	0.173	1503.72	6.554
		1524.43	2.090	1517.35	0.799
		1528.64	1.753	1519.60	3.957

Table S9. Vibrational frequencies for DIMP and $DIMP^{\cdot+}$ with associated mode intensities (see also the next table). Experimental reference [2,5].

Mode	Expt. (cm -1)	Neutral (cm -1)	Int. (km/mol)	Ion (cm -1)	Int.(km/mol)
		3032.07	4.002	3040.83	1.546
		3033.94	4.644	3045.07	1.023
		3037.94	5.551	3052.13	0.243
		3039.44	3.797	3052.60	0.542
		3040.59	2.390	3061.04	3.156
		3049.28	1.576	3081.46	0.405
		3053.68	0.598	3087.67	3.206
		3093.14	1.582	3111.92	5.528
		3098.34	1.146	3114.58	2.946
		3104.67	12.717	3120.21	2.006
		3104.94	10.055	3120.84	1.015
		3107.86	7.901	3126.26	1.167
		3109.50	8.168	3128.10	2.125
		3114.35	8.609	3134.55	6.457
		3126.96	2.730	3136.97	3.988
		3134.94	1.620	3149.32	0.961
		3139.81	0.892	3153.12	0.714
		3032.07	4.002	3040.83	1.546
		3033.94	4.644	3045.07	1.023
CC-H3 str	2872	3037.94	5.551	3052.13	0.243
CC-H3 str	2872	3039.44	3.797	3052.60	0.542
PC-H3 str	2886	3040.59	2.390	3061.04	3.156
C-H str	2902	3049.28	1.576	3081.46	0.405
C-H str	2913	3053.68	0.598	3087.67	3.206
CC-H3 str	2921	3093.14	1.582	3111.92	5.528
CC-H3 str	2921	3098.34	1.146	3114.58	2.946
CC-H3 str	2921	3104.67	12.717	3120.21	2.006
CC-H3 str	2943	3104.94	10.055	3120.84	1.015
CC-H3 str	2945	3107.86	7.901	3126.26	1.167
CC-H3 str	2945	3109.50	8.168	3128.10	2.125
CC-H3 str	2963	3114.35	8.609	3134.55	6.457
CC-H3 str	2963	3126.96	2.730	3136.97	3.988
PC-H3 str	2986	3134.94	1.620	3149.32	0.961
PC-H3 str	2986	3139.81	0.892	3153.12	0.714

Table S10. Continuation of vibrational frequencies for DIMP and DIMP$^{\cdot+}$ with associated mode intensities. Experimental reference [2,5].

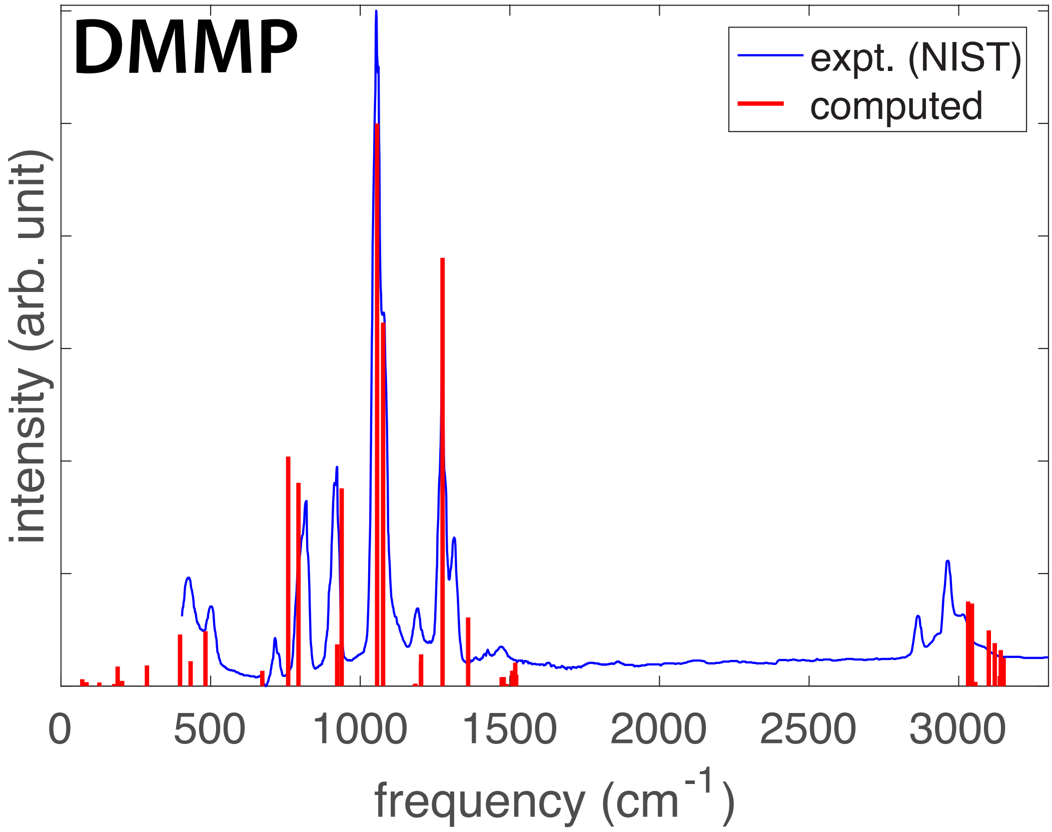

Figure S1. Experimental [6] and computed vibrational spectra of DMMP.

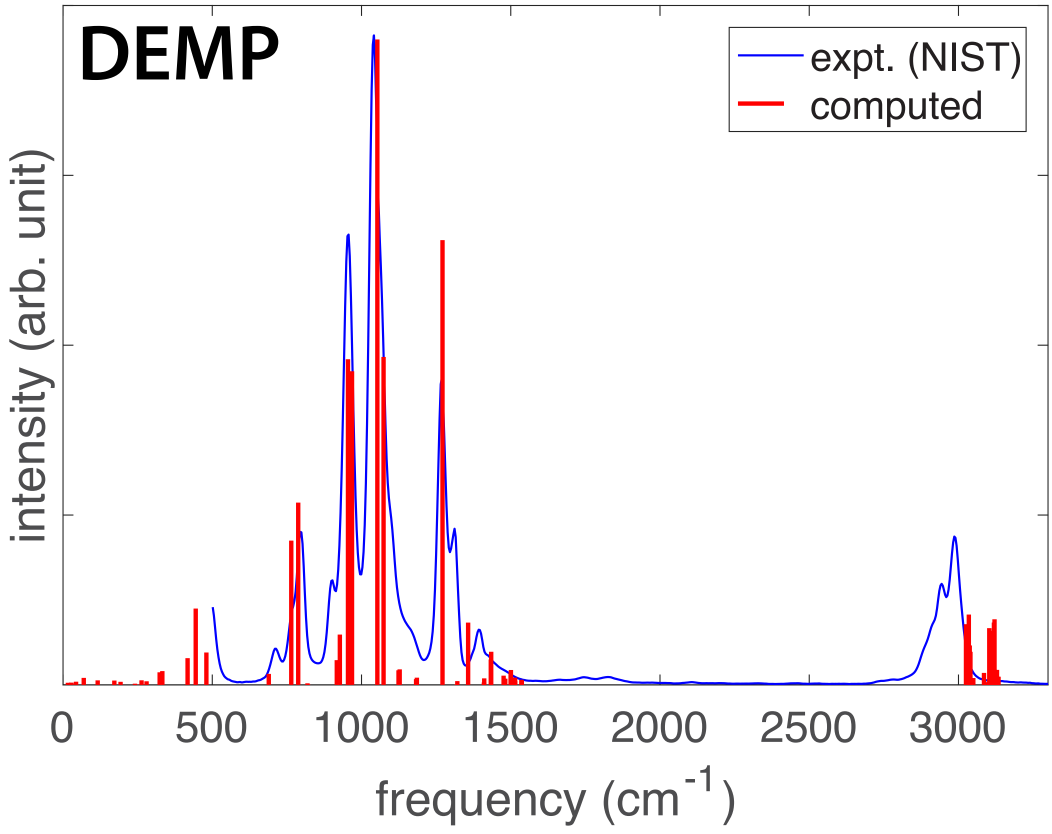

Figure S2. Experimental [6] and computed vibrational spectra of DEMP.

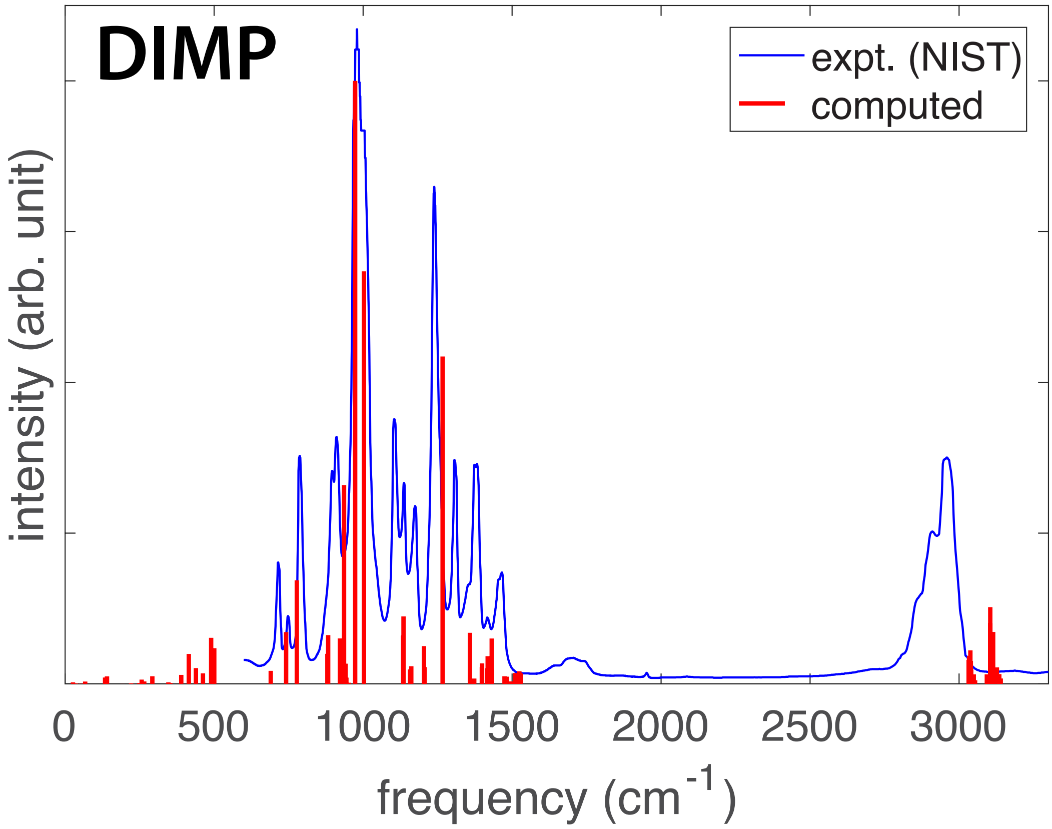

Figure S3. Experimental [6] and computed vibrational spectra of DIMP.

1. Veken, B.J.V.D.; Herman, M.A. VIBRATIONAL SPECTRA OF CH3PO(OCH3)2 AND ISOTOPICALLY SUBSTITUTED DERIVATIVES. *Phosphorus and Sulfur and the Related Elements* **1981**, *10*, 357–367. doi:10.1080/03086648108077388.
2. Hameka, H.F.; Carrieri, A.H.; Jensen, J.O. CALCULATIONS OF THE STRUCTURE AND THE VIBRATIONAL INFRARED FREQUENCIES OF SOME METHYLPHOSPHONATES. *Phosphorus, Sulfur, and Silicon and the Related Elements* **1992**, *66*, 1–11, [https://doi.org/10.1080/10426509208038325]. doi:10.1080/10426509208038325.
3. Ampadu Boateng, D.; Gutsev, G.L.; Jena, P.; Tibbetts, K.M. Ultrafast coherent vibrational dynamics in dimethyl methylphosphonate radical cation. *Phys. Chem. Chem. Phys.* **2018**, *20*, 4636–4640. doi:10.1039/C7CP07261A.
4. Meyrick, C.I.; Thompson, H.W. 53. Vibrational spectra of alkyl esters of phosphorus oxy-acids. *J. Chem. Soc.* **1950**, pp. 225–229. doi:10.1039/JR9500000225.
5. Maarsen, J.W.; Smit, M.C.; Matze, J. The Raman and infra-red spectra of some compounds (iH7C3O)2PXO. *Recueil des Travaux Chimiques des Pays-Bas* **1957**, *76*, 713–723, [https://onlinelibrary.wiley.com/doi/pdf/10.1002/recl.19570760906]. doi:10.1002/recl.19570760906.
6. NIST Standard Reference Database 69. http://webbook.nist.gov/chemistry/. Last checked 12/15/18.
